# Somatic synonymous mutations in regulatory elements contribute to the genetic aetiology of melanoma

**DOI:** 10.1186/s12920-020-0685-2

**Published:** 2020-04-03

**Authors:** Di Zhang, Junfeng Xia

**Affiliations:** 1 College of information science and engineering, Shaoguan University, Shaoguan, Guangdong China; 2 Institutes of Physical Science and Information Technology, Anhui University, Hefei, Anhui China

**Keywords:** Melanoma, Synonymous mutations, Regulatory elements

## Abstract

**Background:**

Non-synonymous mutations altering tumor suppressor genes and oncogenes are widely studied. However, synonymous mutations, which do not alter the protein sequence, are rarely investigated in melanoma genome studies.

**Methods:**

We explored the role of somatic synonymous mutations in melanoma samples from TCGA (The Cancer Genome Atlas). The pathogenic synonymous mutation and neutral synonymous mutation data were used to assess the significance of pathogenic synonymous mutations in melanoma likely to affect genetic regulatory elements using Fisher’s exact test. Poisson distribution probabilities of each gene were used to mine the genes with multiple potential functional synonymous mutations affecting regulatory elements.

**Results:**

Concentrating on five types of genetic regulatory functions, we found that the mutational patterns of pathogenic synonymous mutations are mostly involved in exonic splicing regulators in near-splicing sites or inside DNase I hypersensitivity sites or non-optimal codon. Moreover, the sites of miRNA binding alteration exhibit a significantly lower rate of evolution than other sites. Finally, 12 genes were hit by recurrent potentially functional synonymous mutations, which showed statistical significance in the pathogenic mutations. Among them, nine genes (*DNAH5*, *ADCY8*, *GRIN2A*, *KSR2*, *TECTA*, *RIMS2*, *XKR6*, *MYH1, SCN10A*) have been reported to be mutated in melanoma, and other three genes (*SLC9A2*, *CASR*, *SLC8A3*) have a great potential to impact melanoma.

**Conclusion:**

These findings confirm the functional consequences of somatic synonymous mutations in melanoma, emphasizing the significance of research in future studies.

## Background

In recent years, significant efforts have been made in cancer genomic sequencing by a large consortium. For instance, The Cancer Genome Atlas (TCGA) aims to complete cancer genome sequencing for > 50 types of cancers [1]. The Catalogue Of Somatic Mutations In Cancer (COSMIC) is recognized as the most comprehensive resource for exploring the impact of somatic mutations in cancers, which integrates TCGA and other data source [2]. These data are massive and available but finding the optimal way to mine the underlying information is significant and urgent.

Previous studies have indicated that somatic synonymous mutations are associated with a risk of cancer. Increasing evidence suggests that these synonymous mutations can lead to major splicing defects at the mRNA level or codon usage or function regions annotation features and consequently cause disease [3–6]. Systematic melanoma sequencing studies have identified multiple recurrent mutations that impose functional consequences on the progression of melanoma [7, 8]. These studies mainly focus on non-synonymous mutations, ignoring the functional contribution of synonymous mutations to cancer. Recently, synonymous somatic mutations in *BCL2L12* have been reported to increase BCL2L12 mRNA and protein levels during the development of melanoma [9]. It is important to note that synonymous mutations frequently act as driver mutations by altering splicing and other mechanisms in human cancers [4]. Gotea et al. systematically reviewed the direct impact of synonymous mutations on melanoma and other cancers via splicing, folding, stability, translation and miRNA binding [10]. It is becoming clear that synonymous mutations are not completely neutral, and therefore, their function needs to be further investigated.

**Fig. 1:**
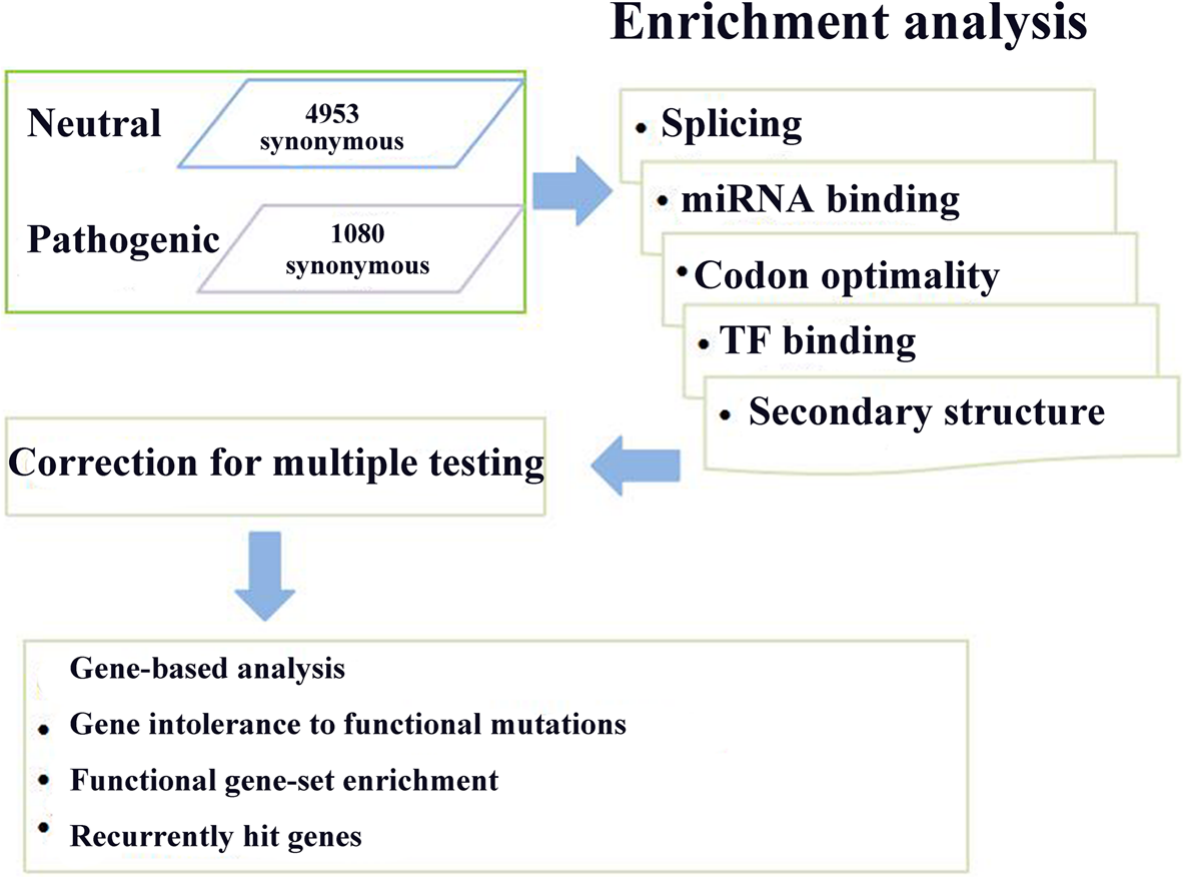
Schematic of the methodology for the analysis of potential functional synonymous mutations in melanoma. The pathogenic synonymous mutation and neutral synonymous data were used to assess the significance of pathogenic synonymous mutations in melanoma likely to affect genetic regulatory elements using Fisher’s exact test

To date, synonymous mutations in melanoma have rarely been investigated in published works. To test whether somatic synonymous mutations can induce the genetic aetiology of melanoma, we mainly focused on five mechanisms: splicing regulation, transcription factor binding, miRNA binding, codon optimality, and the RNA second structure to analyse their impact on melanoma (Fig. 1) [11]. We used the TCGA dataset in COSMIC database [2]. A functional impact score predicted by the FATHMM [12] algorithm was provided in COSMIC. Two groups of somatic synonymous mutations were compared: pathogenic synonymous mutations and the neutral mutations. Our findings present statistical evidence that pathogenic synonymous mutations in melanoma may act through changing exonic splicing regulators in near-splicing sites, altering DNase I hypersensitivity sites, and likely losing optimal codons. We then analysed the recurrent hit genes whose synonymous mutations are likely to contribute to the disease aetiology. We hope that our study will provide a new perspective in understanding somatic synonymous mutations in melanoma from the vast amount of cancer genome sequencing data.

## Results

### Analysis of somatic synonymous mutational patterns in melanoma

**Fig. 2:**
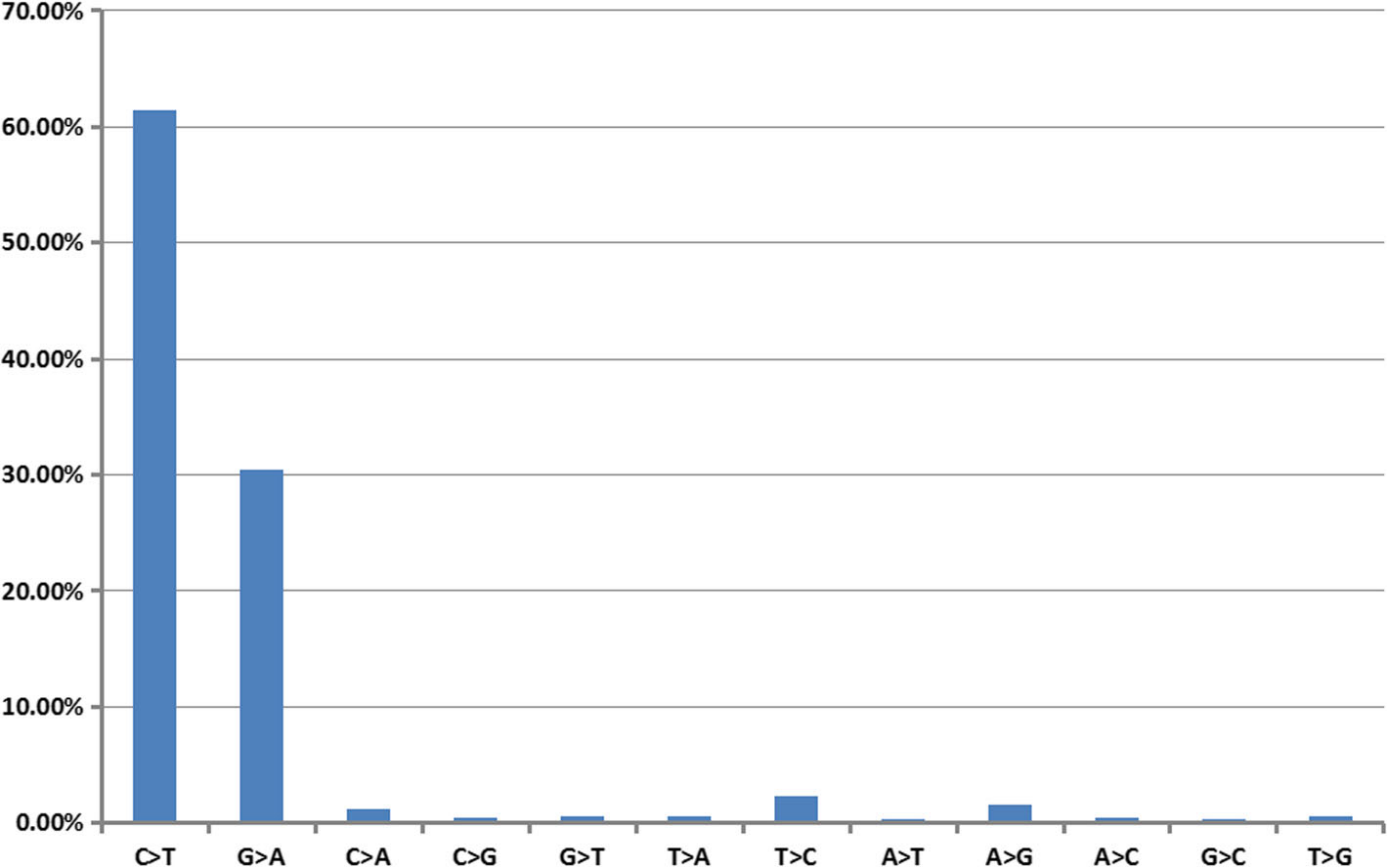
Histogram showing the frequency of occurrence of each of the 12 possible base changes for all synonymous mutations

Somatic synonymous mutations in melanoma were obtained from COSMIC, and only the mutations occur in TCGA samples were obtained for further study. We analysed the distribution of the base changes in the 79,326 somatic synonymous mutations in melanoma. Figure 2 shows that the most frequently occurring base changes were C → T and G → A. Though we have not performed on non-synonymous mutations, but from previous study, we known that they are also the two most frequently observed mutation types in non-synonymous mutations [13]. Two reasons may explain this result. First, the higher frequency of C → T and G → A base changes may be due to the epigenetic modification of DNA that commonly occurs at CpG dinucleotides. Second, the UV radiation (UVR) signature is related to C → T and G → A transitions [14], which is consistent with previously documented findings that UVR exposure can increase melanoma risk [15].

### Pathogenic synonymous mutations in melanoma occur frequently via splicing regulation

As described in the COSMIC, FATHMM scores greater than 0.7 are classified as pathogenic and scores less than 0.5 are labelled as neutral. To increase reliability, we kept only these mutations occurred in at least two samples for pathogenic synonymous mutations. To minimize false positives, we selected only those mutations occurred in one sample for neutral synonymous mutations. As a result of the described procedure, we obtained 1080 pathogenic synonymous mutations and 4953 neutral synonymous mutations in this study.

**Fig. 3:**
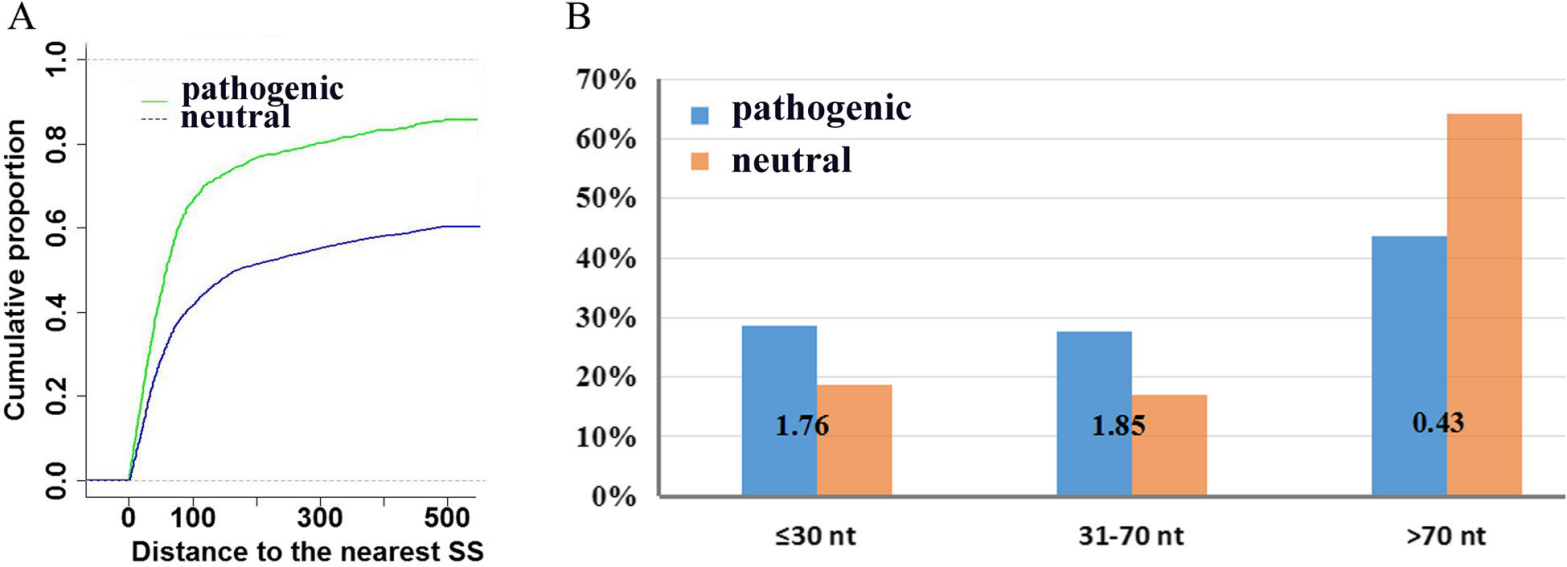
**a** Cumulative proportion curves for the distance to the nearest splice site. The proportion of the synonymous mutations with a distance to the nearest splice sites less than the number indicted in the X axis was plotted on the Y axis. The green line represents the pathogenic synonymous mutations, while the blue line represents the neutral synonymous mutations. **b** Enrichment of synonymous mutations near splice sites (odds ratios shown above)

There has been a long-standing interest in identifying genes involved in the splice site. Recent work specifically described that synonymous mutations within 30 bp of the nearest splice site tended to effect splicing regulation, and this area was defined as the near-splice site [4]. Therefore, in our data, we analysed synonymous mutations within the exonic near-splice site. Our study showed that near-splice sites of synonymous mutations occur nearly twice as often in pathogenic mutations than in neutral mutations (*p* = 5.89 × 10^− 13^, odds ratio [OR] = 1.76, 310 pathogenic mutations, 921 neutral mutations, Fisher’s exact test). This result is inconsistent with Supek’s study [4]. From the cumulative distributions of pathogenic synonymous mutations and neutral synonymous mutations (Fig. 3a), we can see that the distance to the nearest splicing site is closer in the pathogenic mutations than in the neutral mutations. Although Supek’s study reported that the difference in cumulative distributions is near 30 bp [4], in our study, the difference in cumulative distributions between these two classes of synonymous mutations is 31-70 bp (Fig. 3b). This may be because our definition of pathogenic mutations was stricter.

**Fig. 4:**
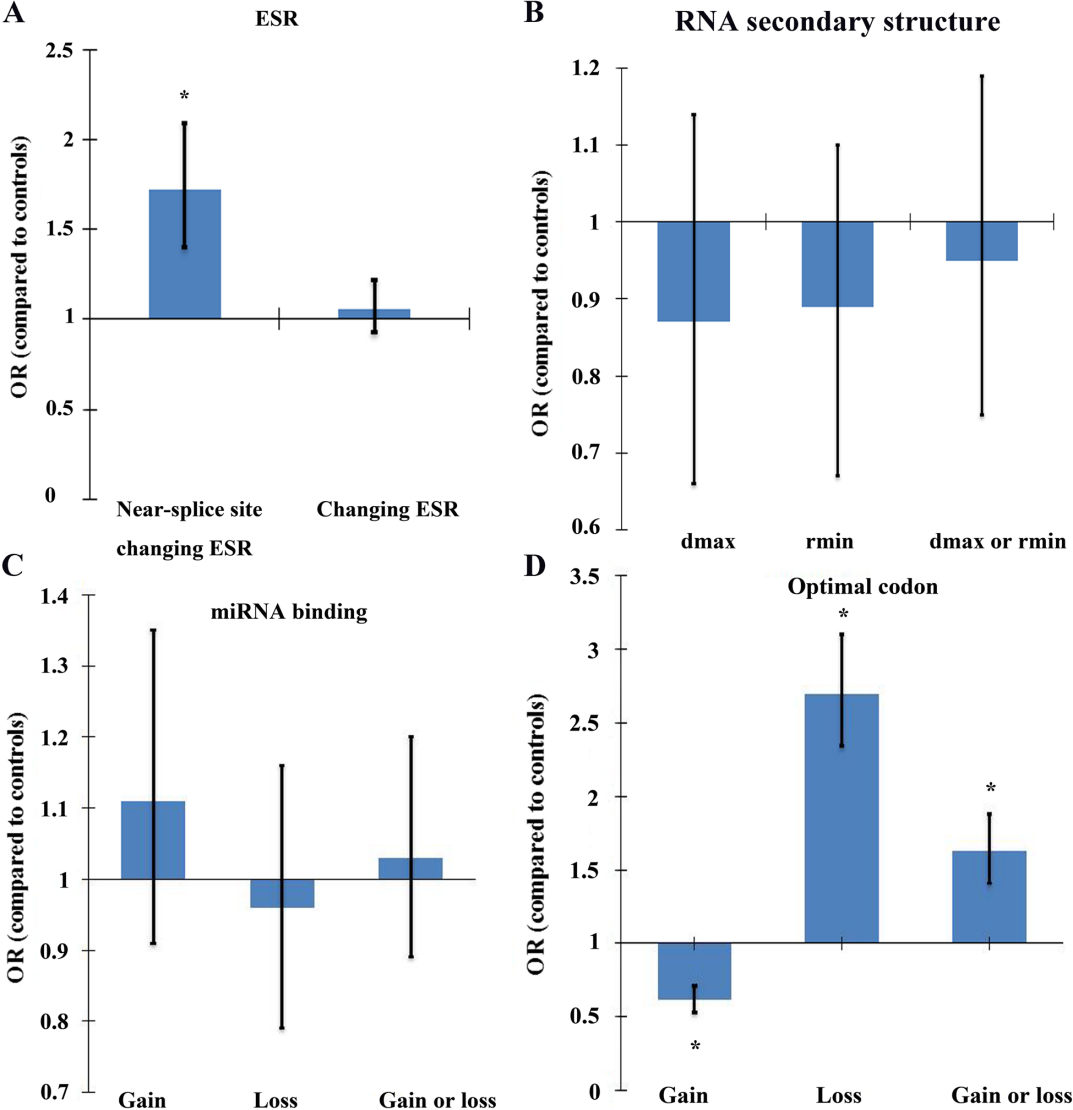
Enrichment analyses of pathogenic synonymous mutations affecting ESR (**a**), RNA secondary structure (**b**), miRNA binding (**c**), and codon optimality (**d**)

Synonymous mutations can influence pre-mRNA splicing by modulating ESS (Exonic Splicing Silencer) and ESE (Exonic Splicing Enhancer) motifs [16]. We referenced the ESE from the RESCUE-ESE website and ESS from the FAS-ESS website (see Methods section). Then, we mapped the synonymous mutations to the list of ESEs and ESSs (402 ESEs and 316 ESSs were collected in our study). From Fig. 4a, we can see that pathogenic synonymous mutations with ESR (exonic splicing regulator) change (i.e., ESE gain, ESS gain, ESE loss, ESS loss) occur in the near-splicing site more often than neutral synonymous mutations (*p* = 1.28 × 10^− 7^, OR = 1.72, Fisher’s exact test). This in support of the research that pathogenic synonymous mutations are deleterious owing to disrupting ESR sequences, with a direct result in aberrant gene splicing [17].

### Pathogenic synonymous mutations in melanoma do not frequently alter mRNA secondary structure or miRNA target sites

Next, we consider whether pathogenic synonymous mutations contribute to melanoma by influencing the mRNA secondary structure, which also changes translation efficiency [18]. We used RNAsnp software, which uses the local regions of maximal structural change between the mutant and wild-type to predict the synonymous mutation effects on mRNA secondary structure [19]. However, we found that pathogenic synonymous mutation effects on mRNA folding do not seem to be common mechanisms in melanoma (see Fig. 4b). While most common mutation associated with cystic fibrosis affecting the mRNA secondary structure [10]. It remains to be determined whether pathogenic synonymous mutations affect mRNA secondary structures depending on disease category.

Synonymous mutations interfere with miRNA binding, which can alter the nucleotide sequences targeted by miRNAs [10]. However, we found that the pathogenic synonymous mutations effects on miRNA binding do not seem to be common mechanisms in melanoma (see Fig. 4c). The nucleotide sequences of the putative miRNA binding site exhibit a significantly lower rate of evolution at the synonymous mutation sites than other gene sequences [20]. In our study, we considered whether the site of miRNA binding alteration presents high conservation. As expected, the conservation of the site with miRNA binding alteration is higher than the site with no miRNA binding alteration (*p* = 0.028) in pathogenic synonymous mutations. Therefore, pathogenic synonymous mutations do not seem to affect RNA secondary structure or miRNA binding sites, although they may still be vital in individual cases [9].

### Pathogenic synonymous mutations in melanoma decrease codon optimality

Pathogenic synonymous mutations affect gene activity through diverse molecular mechanisms [21]. We evaluated whether pathogenic synonymous mutations in melanoma tend to increase or decrease the use of optimal codons, thereby affecting translation efficiency [22]. When a synonymous mutation changes an optimal codon to a non-optimal codon, it may introduce a pause in the translation machinery. Then, a pathological condition will be decided by the degree of the affected functional protein. As expected, pathogenic synonymous mutations show a general tendency towards less optimal codons, while gain of the optimal codons is seen in neutral synonymous mutations (Fig. 4d).

### Pathogenic synonymous mutations in melanoma are likely located in DNase I hypersensitive sites (DHSs)

Functional mutations are more likely to target transcription factor (TF) binding sites and are especially located in DNase I hypersensitive sites (DHSs), which are open chromatin regions for proteins to approach [11]. Chromatin regions of DHSs and TF are functionally related to transcriptional activity and necessary for the binding of proteins such as transcription factors. According to genome-wide association studies, DHSs are significantly enriched for transcriptional signals [23]. We downloaded the TF binding site and DHS information from the ENCODE (Encyclopedia of DNA Elements) experiment matrix. We analysed whether pathogenic synonymous mutations in melanoma was preferentially found in TF and DHS and found a significant target in DHS but no significant target in TF compared to the neutral synonymous mutations.

### Potentially functional synonymous mutations in melanoma are involved in transport pathways

**Fig. 5:**
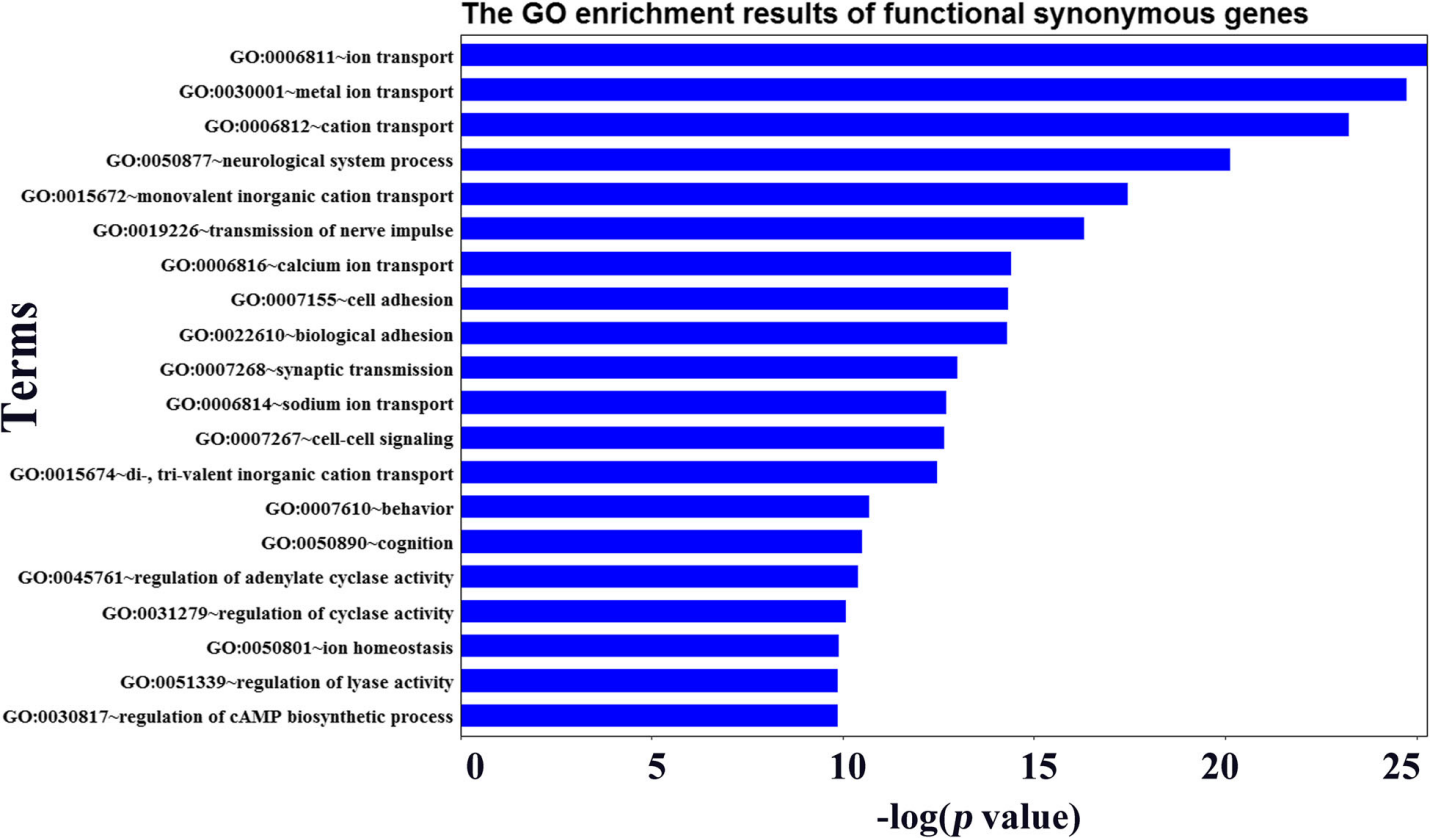
The biological functions significantly enriched by genes with functional synonymous mutations. The top 20 GO terms with *p* values < 0.05 are displayed in ascending order

Through the above analyses, we found that the pathogenic synonymous mutations are enriched in three aspects, including exonic splicing regulators within the near-splicing site, DNase I hypersensitivity sites, and loss of optimal codons. If one of the pathogenic synonymous mutations is in accord with one of the three aspects, we defined it as a potentially functional synonymous mutation. Among the 1080 pathogenic synonymous mutations, 713 mutations were functional synonymous mutations, which presents 11.8% (713/6033) of total synonymous mutations (1080 pathogenic and 4953 neutral synonymous mutations) used in this study. Functional gene enrichment analysis was performed using the online service website DAVID [24]. As shown in Fig. 5, these genes were significantly enriched in genes associated with transport pathways (e.g., “metal ion transport”, Benjamini-Hochberg corrected *p* value [p_BH_] =2.48 × 10^− 8^; “cation transport”), neuronal function (e.g., “neurological system process”, p_BH_ = 6.45 × 10^− 8^), and signalling pathways (e.g., “cell-cell signalling”, p_BH_ = 6.25 × 10^− 4^). In recent years, it has become increasingly clear that ion transporters (such as GO:0006811~ion transport, seen in Fig. 5) is an important mechanism in the development of drug resistance [25]. We infer that the transport pathway may be related to drug resistance, and suggest that they may have strong potential as diagnostic and therapeutic targets in melanoma treatment.

### Candidate genes that are recurrently hit by potentially functional synonymous mutations in melanoma

The identification of these functional synonymous mutations based on high recurrence can assist in detecting new genes that participate in melanoma. In our study, we assessed the statistical significance of observing potential functional synonymous mutations for each gene by analysing high-quality variant calls across 60,706 human exomes [26]. Previous research indicates that the genome-wide significance threshold is 2.74 × 10^− 6^ [27]. In our study, the probability of observing potential functional synonymous mutations was calculated by Poisson distribution. Table 1 shows significant genes with multiple synonymous mutations across 341 melanoma patients. Notably, not all genes with many synonymous mutations have significant *p* values. For example, *TTN* has 5 potential functional synonymous mutations in the case group but does not have a nominally significant *p* value (*p* value = 7.11 × 10^− 6^), owing to the long length of the gene. Among these genes identified in this study, 12 genes are hit by potential functional synonymous mutations in melanoma (Table 1). Of these, *DNAH5* mutations in melanoma samples correlate with a negative outcome [28]. *ADCY8* mutations were previously identified in melanoma patients [29]. *KSR2* have been shown to have pro-tumorigenic roles [30].*GRIN2A* harbors 5 potential functional synonymous mutations and encodes the NMDA (N-methyl-(D)-aspartic acid) receptor subunit, which bears the agonist binding site for glutamate [31]. It has been reported that 11 somatic mutations in GRIN2A were involved in melanoma [32]. The *p* value (9.99 × 10^− 12^) for this gene is less than the genome-wide significance threshold. *TECTA* has significantly more potential functional synonymous mutations than expected (*p* = 1.78 × 10^− 8^), in which mutations occurred in melanoma [33]. In our study, for the *TECTA* gene, 2 mutations were found in the near-splice site and accompanied by ESR changes, and 2 mutations were found in DHSs. In addition, both *GRIN2A* and *TECTA* were among the most intolerant genes according to the RVIS (Residual Variation Intolerance Score), indicating that the variants within these genes are under natural selection and may be more likely to influence melanoma. *RIMS2* significantly upregulated in monosomy 3 melanomas [34]. *XKR6* mutations already reported in cutaneous malignant melanoma cell lines [35]. *SCN10A* commonly mutated among TCGA melanoma samples [28]. *MYH1* has recently been shown to be upregulated in melanoma [36]. We also identified mutations in three genes (*SLC9A2*, *CASR*, *SLC8A3*) never reported in melanoma, which might deserve further investigations.

**Table 1:** Genes with recurrent synonymous mutations potentially affecting regulatory elements

Gene	Gene length	Putative mechanisms^a^	Number of functional synonymous mutations	Number of expected synonymous mutations	*p* values
*DNAH5*	13,875	disrupt ESR sequence in near SS, alter DHS, and decrease the use of optimal codons	13	0.037106647	3.92 × 10^−29^
*ADCY8*	3756	disrupt ESR sequence in near SS, alter DHS, and decrease the use of optimal codons	5	0.014354406	5.02 × 10^−12^
*GRIN2A*	4395	disrupt ESR sequence in near SS, alter DHS, and decrease the use of optimal codons	5	0.01648022	9.99 × 10^−12^
*SLC9A2*	2439	disrupt ESR sequence in near SS, alter DHS, and decrease the use of optimal codons	4	0.008004311	1.7 × 10^−10^
*CASR*	3267	alter DHS, decrease the use of optimal codons	4	0.011093165	6.25 × 10^−10^
*KSR2*	2853	disrupt ESR sequence in near SS, alter DHS, and decrease the use of optimal codons	4	0.012140187	8.96 × 10^−10^
*MYH1*	5820	disrupt ESR sequence in near SS, alter DHS, and decrease the use of optimal codons	4	0.014354315	1.75 × 10^−9^
*TECTA*	6468	disrupt ESR sequence in near SS, alter DHS, and decrease the use of optimal codons	4	0.02571247	1.78 × 10^−8^
*SLC8A3*	2784	disrupt ESR sequence in near SS, decrease the use of optimal codons	3	0.00836151	9.68 × 10^−8^
*RIMS2*	4050	alter DHS, decrease the use of optimal codons	3	0.009850803	1.58 × 10^− 7^
*XKR6*	1926	alter DHS	3	0.010850219	2.11 × 10^−7^
*SCN10A*	5871	alter DHS, decrease the use of optimal codons	3	0.016168958	6.96 × 10^−7^

^a^*ESR:* exonic splicing regulators, *SS:* splicing sites, *DHS:* DNase I hypersensitivity sites

## Discussion

The first theme of this study is to investigate somatic synonymous mutations in regulatory elements contribute to the genetic aetiology of melanoma. From our analyses, we found that mutations with near-splicing site mutations changing ESR, targeting DHI sites and preferentially using non-optimal codons are significantly enriched in pathogenic synonymous mutations. After performing gene functional enrichment, we obtained several potential functional synonymous mutations related to biological processes such as transporter and signalling pathway that have previously been implicated in melanoma. Pathogenic synonymous mutations may participate in transport pathway and might be related to drug resistance.

Another theme of this study is the estimation of the number of genes in which functional synonymous mutations affecting regulatory elements in melanoma. Biases in functional mutation could make some tissues more prone to accumulating functional mutations in certain genes (e.g., exposure to mutational processes). By comparing the number of expected synonymous mutations, we inferred 12 candidate genes across melanoma. Most of these genes generally agree with previously reported genes in melanoma. Remarkably, there is a connection between candidate genes and intolerant gene under positive selection. Recent analysis of synonymous and non-synonymous mutations in tumor genomes has revealed that positive selection accelerates the fixation of somatic mutations in a gene-specific manner [37, 38]. Our results imply that selection pressures during melanoma progress is a vital event.

The excesses of potential functional synonymous mutations across gene sets were evaluated by calculating the significance for individual genes. Though the potential functional synonymous mutations take a small part of synonymous mutations, a few mutations tend to drive melanoma. It is likely that pathogenic synonymous mutations affect regulatory elements. These mutations can explain a substantial part of melanoma liability.

## Conclusion

In summary, we used existing published TCGA data to identify pathogenic synonymous mutations in melanomas. Future functional and preclinical studies are needed to be conducted to determine whether these synonymous mutations are pathogenic or neutral mutations. Going forward, the study presented here may pave the way for analysing synonymous mutation data in other types of cancer, like lung cancer.

## Methods

### Data collection

Published somatic synonymous mutations in melanoma were collected from the TCGA data in COSMIC (v80 release). For gene with multiple transcripts, only the longest transcript was remained. The FATHMM (Functional Analysis Through Hidden Markov Models) scores predicting functional consequences of somatic synonymous mutations were also obtained from the COSMIC.

### Analysis of synonymous mutations changing splicing regulation

The annotation for the distance to the nearest splice site for each synonymous mutation was obtained by SeattleSeq annotation 138 online website (http://snp.gs.washington.edu/SeattleSeqAnnotation138/).

To investigate whether the near-splice site synonymous mutations tend to affect exonic splicing regulators (exonic splicing enhancer (ESE) and silencer (ESS)), we used the following lists of predicted ESEs and ESSs: 1) top 200 ESEs with “ESEseq” descending order and top 200 ESSs with “ESSseq” ascending order in the attachment of literature, in which ESE and ESS were experimentally tested [39]. 2) A total of 238 ESEs were curated from RESCUE-ESE Web Server (http://hollywood.mit.edu/burgelab/rescue-ese), which provides a hybrid computational/experimental method to identify candidate ESEs in vertebrate exons. 3) A total of 176 ESSs were curated from FAS-ESS Web Server (http://hollywood.mit.edu/fas-ess), which uses a splicing reporter system to identify ESSs. The list of ESEs was the union set of the above two resources, and similarly, the list of ESSs was the union set of the above two resources (in total, 402 ESEs and 316 ESSs). We compared the set of sequences consisting of the mutated site with 6 bp windows (− 5~0, − 4~ + 1, 3~ + 2, 2~ + 3, 1~ + 4, and 0~ + 5 when the mutation site is at position 0) to the reference lists of ESE and ESS sequences, and checked whether each synonymous mutation caused ESE or ESS events. The reference site was calculated similarly. Four types (ESE gain, ESE loss, ESE gain or ESS loss) of ESR change were considered for all synonymous mutations.

SPANR (Splicing-based Analysis of Variants) was adopted to measure the impact of synonymous mutations on splicing regulation [40] and evaluations were performed with all the synonymous mutations.

### DNase I hypersensitive sites (DHS) analysis

The mapped read BAM files in Encyclopedia of DNA Elements (ENCODE) were downloaded from http://genome.ucsc.edu/ENCODE/downloads.html, which were used for DHS annotations. The raw downloaded DHS data contains the defining DHS peaks and footprints. Synonymous mutations that lied near DHS sites were not considered as overlapping with DHS.

### miRNA binding site analysis

The FASTA file of high confidence miRNAs was downloaded from miRBase (http://www.mirbase.org/blog/2014/07/high-confidence-mirna-set-available-for-mirbase-21/). Then, we extracted the miRNA seed sequences. When the first base was adenine, position 2–7 sequences were selected. Base A appears to anchor the very 3′ terminus of the miRNA complementary site, which suggests that requiring a 6-nt seed match followed by this “A anchor” would increase the specificity of target prediction [41]. Otherwise, position 2–8 sequences were selected. We compared the corresponding mutation sequences (7-bp windows (− 6~0, − 5~ + 1, 4~ + 2, 3~ + 3, 2~ + 4, − 1~ + 5, and 0~ + 6 when the mutation site is at position 0) and the miRNA seed sequences, and evaluated whether a synonymous mutation can cause at least on loss or gain in the miRNA binding site.

### Optimal codon analysis

To study whether synonymous mutations lead to a gain or loss of the optimal codon events, we downloaded the previously defined optimal codons from Table S6 of literature [4]. For each synonymous mutation, the corresponding codons for reference and variant alleles were obtained using the variant effect predictor tool [42].

### RNA secondary structure analysis

To evaluate the impact of synonymous mutations on RNA secondary structures, we input sequence files and lists of mutations to be tested into the RNAsnp software [19] with default parameters. An option to run RNAsnp on the genome sequence is to split the input sequence chromosome-wise, which can be done similarly for the synonymous mutations (without chromosome number as prefix). The maximum Euclidean distance and minimum Pearson correlation coefficient were the two methods used to calculate the *p* value.

### Mutations in genes intolerant to functional variation analysis

The genes intolerant to functional variation were downloaded from the literature [43]. We considered the top 25% percentage of the genes as “intolerant” genes. Thus, mutations corresponding to these intolerant genes have RVIS (Residual Variation Intolerance Score) information. Mutations without RVIS information were excluded from the analysis.

### Evolutionary conservation analysis

Phylop.mammalian conservation scores for the variant were obtained from MyVariant.info [44]. This score is positively correlated with evolutionary conservation.

### Gene functional enrichment analysis for the genes with potential functional synonymous mutations

Genes with potential functional synonymous mutations were input into the DAVID online web server (V6.7) [24]. The biological process was the category of functional annotations. B-H was used to adjust the *p* value. Terms with ≤5 hit counts in the query list were filtered.

### Recurrent gene analysis

To mine the genes with multiple potential functional synonymous mutations affecting regulatory elements, we downloaded the gene’s corresponding probability of synonymous mutations according to a recently developed pipeline for the interpretation of synonymous mutations [27]. The number of expected synonymous mutations *λ* in each gene is calculated as follows:

$$ \lambda =2\ast R\ast S, $$

where *R* represents the mutation rate for each synonymous mutation [26], *S* represent the number of samples (341 samples in our study).

Poisson distribution probabilities of each gene *P* was calculated as follows [27]:

$$ \mathrm{P}\left(\mathrm{X}=\upkappa \right)=\frac{\lambda^{\kappa }}{\kappa !}{e}^{-\lambda }, $$

where *k* represents the number of functional synonymous mutations in corresponding gene, which was decided by the number of functional synonymous mutations in the gene. Only the Poisson distribution probabilities *p* of each gene less than 1 × 10^− 6^ was defined as recurrent genes. The *p*-values were not adjusted for multiple comparisons.

## Data Availability

The datasets analysed during the current study are available in the COSMIC database (https://cancer.sanger.ac.uk/cosmic), ENCODE datasets (http://genome.ucsc.edu/ENCODE), and miRBase database (http://www.mirbase.org). All the other datasets analysed during this study are openly available online and URLs are provided in this published article.

## Abbreviations

COSMIC: The Catalogue Of Somatic Mutations In Cancer
DHSs: DNase I hypersensitive sites
ENCODE: Encyclopedia of DNA Elements
ESE: Exonic Splicing Enhancer
ESR: Exonic Splicing Regulator
ESS: Exonic Splicing Silencer
FATHMM: Functional Analysis through Hidden Markov Models
RVIS: Residual Variation Intolerance Score
TCGA: The Cancer Genome Atlas
TF: Transcription Factor
